# Recurrent variation in the active NOR sites in the monkey frogs of the genus *Pithecopus* Cope, 1866 (Phyllomedusidae, Anura)

**DOI:** 10.3897/CompCytogen.v13i4.37687

**Published:** 2019-10-21

**Authors:** Joana Moura Gama, Camilla Borges Gazolla, Deborah Yasmin de Souza, Shirlei Maria Recco-Pimentel, Daniel Pacheco Bruschi

**Affiliations:** 1 Programa de Pós-Graduação em Genética (PPG-GEN). Departamento de Genética, Universidade Federal do Paraná (UFPR), Setor de Biológicas, Av. Coronel Francisco H. Dos Santos, 100, 81531-980, Curitiba, PR, Brazil Universidade Federal do Paraná Curitiba Brazil; 2 Departamento de Biologia Estrutural and Funcional, Universidade Estadual de Campinas (UNICAMP), Cidade Universitária, 13083-863, Campinas, SP, Brazil Universidade Estadual de Campinas Campinas Brazil

**Keywords:** chromosomal evolution, *
Pithecopus
*

## Abstract

Treefrogs of the genus *Pithecopus* Cope, 1866 exhibit expressive chromosomal homogeneity which contrasts with a high variation frequency of the nucleolus organizer region (NOR) related to the group. Currently, the genus contains eleven species and no chromosomal data are available on *P. palliatus* Peters, 1873, *P. ayeaye* Lutz, 1966 and *P. megacephalus* Miranda-Ribeiro, 1926. Here, we describe the karyotypes of these three species based on Giemsa staining, C-banding, silver impregnation and in situ hybridization (FISH). We were also analyze the evolutionary dynamic of the NOR-bearing chromosome in species of genus under a phylogenetic view. The results indicate that *P. palliatus*, *P. ayeaye*, and *P. megacephalus* have similar karyotypes, which are typical of the genus *Pithecopus*. In *P. palliatus* the NOR was detected in the pericentromeric region of pair 9p whereas in *P. ayeaye* and *P. megacephalus* we report cases of the multiple NOR sites in karyotypes. In *P. ayeaye* the NOR was detected in the pericentromeric region of pair 9p in both homologues and additional sites was detected in pairs 3q, 4p, and 8q, all confirmed by FISH experiments. Already in *P. megacephalus* the NOR sites were detected in pericentromeric region homologues of pair 8q and additionally in one chromosome of pair 13q. A comparative overview of all the *Pithecopus* karyotypes analyzed up to now indicates the recurrence of the NOR-bearing chromosome pairs and the position of the NORs sites on these chromosomes. We hypothesized that this feature is a result of a polymorphic condition present in the common ancestor of *Pithecopus*. In such case, the lineages derived from polymorphic ancestor have reached fixation independently after divergence of lineages, resulting in a high level of homoplasy observed in this marker. Our findings help to fill the gaps in the understanding of the karyotype of the genus *Pithecopus* and reinforce the role of the evolutionary dynamics of the rDNA genes in karyotype diversification in this group.

## Introduction

Duellman et al. (2016) recognized the genus *Pithecopus* Cope, 1986 (the monkey frogs) as a distinct taxon from the genus *Phyllomedusa* Wagler, 1930, with which it had previously been synonymized, and Frost (2019) concluded that the genus contains 11 valid species. The genus is distributed throughout Central America from east of the Andes and northern Argentina (Frost 2019). Molecular inferences (Faivovich et al. 2010; Bruschi et al. 2014; Duellman et al. 2016; Haga et al. 2017) have recovered two well-supported clades in *Pithecopus* with a strong biogeographic component. One clade includes primarily lowland species (*Pithecopus azureus* Cope, 1862, *Pithecopus araguaius* Haga, Andrade, Bruschi, Recco-Pimentel & Giaretta, 2017, *Pithecopus hypochondrialis* Daudin, 1800, *Pithecopus palliatus* Peters, 1873 and *Pithecopus nordestinus* Caramaschi, 2006), while the second clade encompasses species that inhabit highland regions and plateaus (*Pithecopus ayeaye* Lutz, 1966, *Pithecopus centralis* Bokermann, 1965, *Pithecopus megacephalus* Miranda-Ribeiro, 1926, *Pithecopus oreades* Brandão, 2002, and *Pithecopus rusticus* Bruschi, Lucas, Garcia & Recco-Pimentel, 2014), with the exception of *Pithecopus rohdei* Mertens, 1926, which is distributed throughout the altitudinal gradient of the Brazilian Atlantic Forest. Interestingly, high levels of endemism (Magalhães et al. 2018) and cryptic diversity (Faivovich et al. 2010, Ramos et al. 2019) have been reported in the “highland” clade. Cytogenetic data have already indicated interpopulational variability in *P. rohdei* (Barth et al. 2009, Paiva et al. 2009, Bruschi et al. 2012), which could be the first step to speciation. Population genetic divergence was recently confirmed by a molecular analysis using nuclear and mitochondrial markers (Ramos et al. 2019), which emphasizes the potential contribution of karyotype data as complementary evidence for the identification of cryptic diversity.

No published chromosomal data are available on *P. palliatus*, *P. ayeaye*, and *P. megacephalus*. *Pithecopus palliatus* is a member of the lowland clade (Faivovich et al. 2010, Duellman et al. 2016), and inhabits temporary pools in the tropical rainforests of the upper Amazon basin in Ecuador, Peru, northern Bolivia and western Brazil (Frost 2018). By contrast, *P. ayeaye* and *P. megacephalus* have more restricted geographic ranges in southeastern Brazil, where they form small, highly structured and isolated populations with a discontinuous distribution in mountaintop isolates (“sky islands”) in highland Rockfield (“*campo rupestre*”) ecosystems (Magalhães et al. 2018, Ramos et al. 2018).

*Pithecopus ayeaye* is endemic to high altitudes in southeastern Brazil. This species is listed as critically endangered (CR) by the International Union for Conservation of Nature, IUCN (Caramaschi et al. 2016), although reports of new occurrence localities (Araújo et al. 2007, Baêta et al. 2009) led to the removal of the species from the Brazilian List of Endangered Species (ICMBio 2014). Magalhães et al. (2018) recently identified three different evolutionary significant units (ESUs) of *P. ayeaye* in distinct *campo rupestre* ecosystems using multilocus DNA sequences and emphasized the need for the inclusion of the genetic profile of this species in the definition of regional conservation policies.

*Pithecopus megacephalus* occurs at high elevations (above 800 m a.s.l.) in the *campo rupestre* systems of the Southern Espinhaço Mountain Range (Oliveira et al. 2012). Using multilocus analyses, Ramos et al. (2018) found considerable genetic structuring among three *P. megacephalus* populations from different “sky islands” in the Espinhaço Range, and evidence of low gene flow among these populations.

Here, we advance our understanding of the cytogenetics of the genus *Pithecopus* and compile the karyotype data available on the genus to discuss its chromosomal features from a phylogenetic perspective.

## Material and methods

### Biological samples

We analyzed populations of *P. ayeaye*, *P. megacephalus* and *P. palliatus* sampled in Brazilian localities (Table 1). Specimen collection was authorized by the Biodiversity Information System (SISBIO) of the Chico Mendes Institute for Biodiversity Conservation (ICMBio), through license 45183-3. Voucher specimens were deposited in the “Prof. Dr. Adão José Cardoso” Museum of Zoology (**ZUEC**) at University of Campinas (**UNICAMP**), in São Paulo state, Brazil.

**Table 1.** Details of the *Pithecopus* species and specimens sampled for the cytogenetic analyses presented in this study.

Species	Number of specimens	Locality/State^1^	Geographic coordinates	ZUEC^2^ number
*P. ayeaye*	03 ♂	Brumadinho/MG	20°29'S, 44°19'W	16403–16405
*P. megacephalus*	03 ♂	Santana do Riacho/MG	19°10'S, 43°42'W	In the accept
*P. palliatus*	12 ♂ + 3 ♀	Boca do Acre/AM	8°44'S, 67°23'W	17037–17051

^1^AM = Amazonas; MG = Minas Gerais; ^2^ZUEC = “Prof. Dr. Adão Cardoso” Museum of Zoology at University of Campinas (UNICAMP).

### Cytogenetic analyses

Metaphase cells were obtained from the intestines and testes of animals previously treated with 2% colchicine (*Sigma – Aldrich*; 0.02 ml per 1 g of body weight), following procedures modified from King and Rofe (1976) and Schmid (1978). Prior to the removal of the organs, the animals were anesthetized profoundly with 5% Lidocaine, applied to the skin, to minimize suffering, as recommended by the Herpetological Animal Care and Use Committee (HACC) of the American Society of Ichthyologists and Herpetologists (available at http//www.asih.org/publications). The chromosome preparations were stained with 10% Giemsa and C-banded (Sumner 1972). The C-banded chromosomes of *P. ayeaye* were stained with fluorochrome AT-specific DAPI and GC-specific Mytramycin (MM).

The nucleolus organizer regions (NOR) were revealed by the silver nitrate impregnation technique (Ag-NOR) following Howell and Black (1980). Fluorescent *in situ* hybridization (FISH) was used to confirm the presence of multiple NORs in the *P. ayeaye* karyotype. The FISH assays followed the protocol of Viegas-Péquignot (1992). The 28S rDNA probe were isolated from *Pithecopus hypochondriasis*, cloned and sequenced by Bruschi et al (2012) and sequence is available in GenBank database under accession number HM639985. The probe was labeled with digoxigenin 11-dUTP (Roche Applied Science). The hybridized signals were detected using an anti-digoxigenin antibody conjugated with rhodamine (600 ng/mL) and counterstained with 0.5 mg/ml of DAPI.

We analyzed twenty metaphase plates per individual for each of the applied methods. The metaphases were photographed under an Olympus microscope and analyzed using the Image Pro-Plus software, version 4 (Media Cybernetics, Bethesda, MD, USA). The chromosomes were ranked and classified according to the scheme of Green and Sessions (1991).

## Results

**Figure 1.**
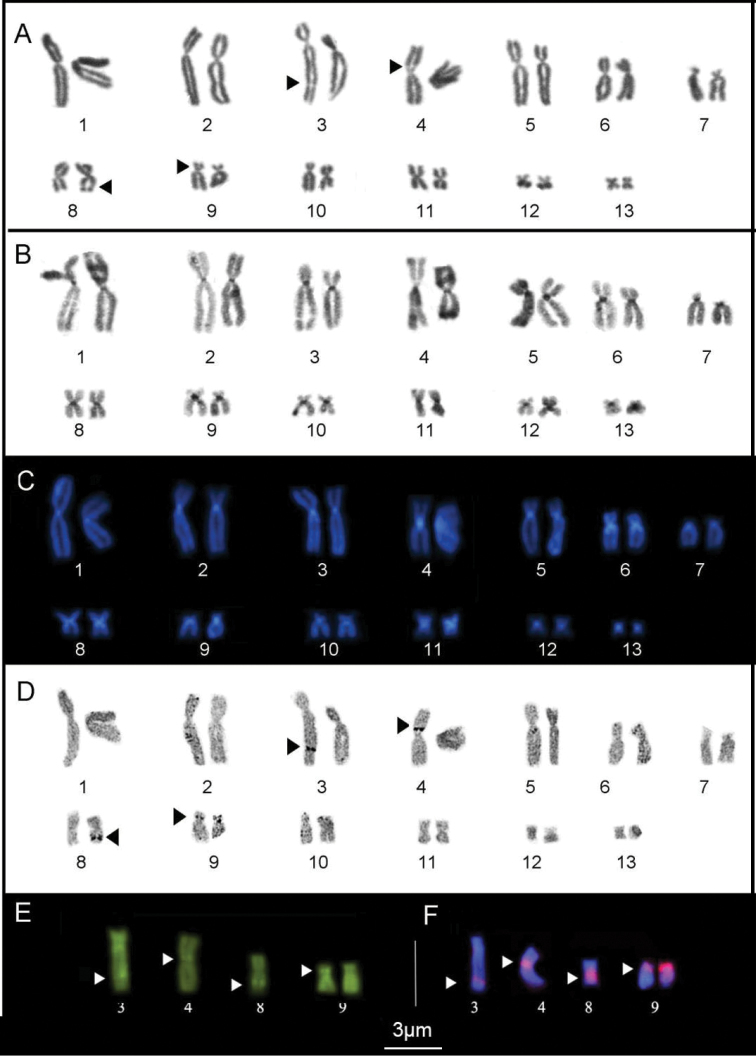
Karyotype of *P. ayeaye* prepared by conventional Giemsa staining (**A**) C- banding (**B**) Ag-NOR (**D**) DAPI staining after C-banding (**C**). Chromosomes submitted to Mytramicim (MM) (**E**) and FISH experiments with a nucleolar 28S rDNA probe (**F**). The arrow indicates indicates secondary constrictions; the arrowheads indicate multiple NOR site.

**Figure 2.**
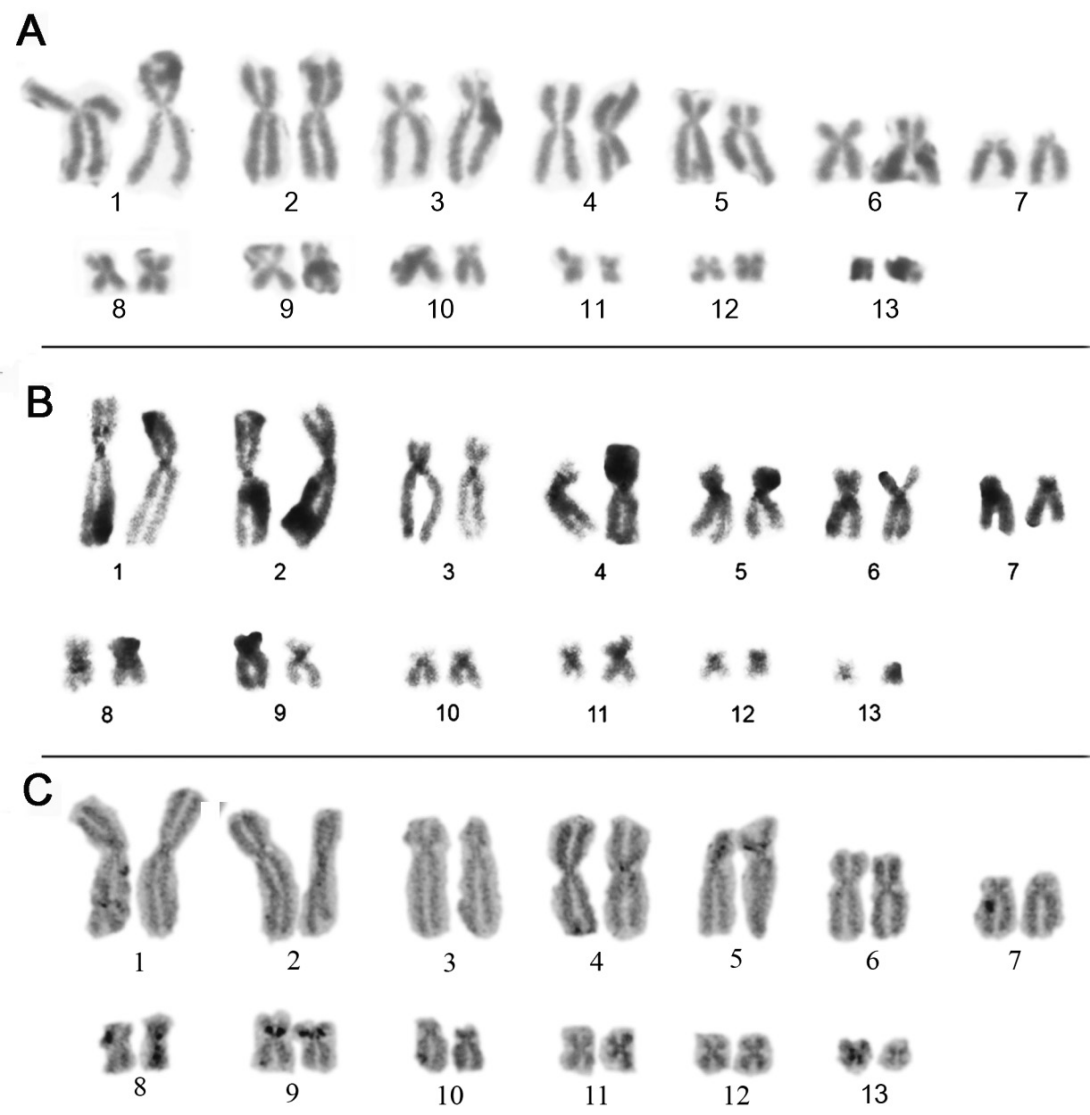
Karyotype of *P. palliatus* prepared by conventional Giemsa staining (**A**) C-banding (**B**) and Ag-NOR method (**C**). Secondary constrictions observed coincided with the Ag-NOR sites (**C**).

**Figure 3.**
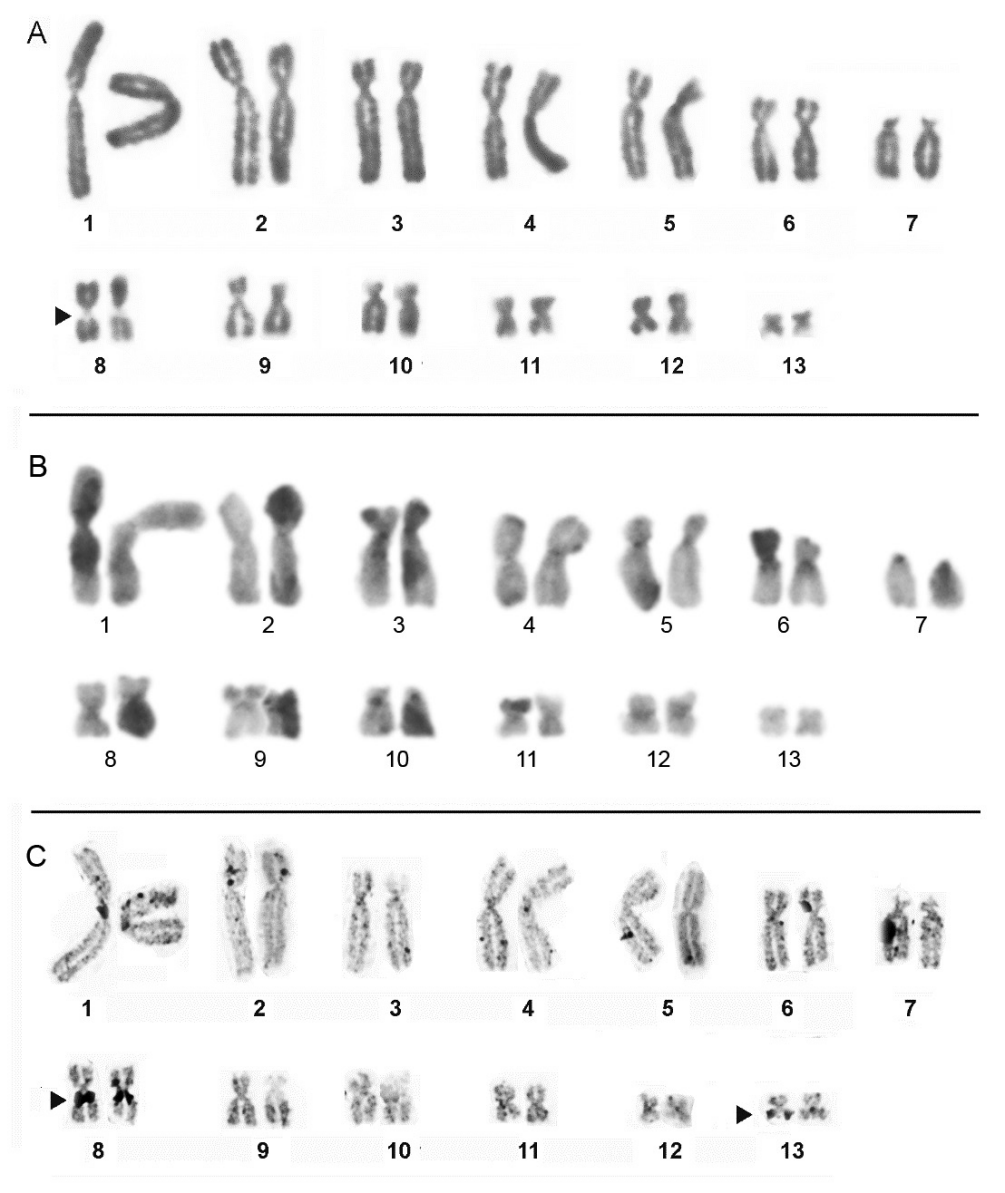
Karyotype of *P. megacephalus* prepared by conventional Giemsa staining (**A**) C-banding (**B**) and Ag-NOR method (**C**). The arrow indicates secondary constrictions in the pair 8 correspond to NOR sites. Note the additional NOR in one homologue of pair 13.

All three species analyzed here had a diploid number of 26 chromosomes. The chromosomal complement of all three species (Figs 1A, 2A, and 3A) consisted of the four metacentric pairs (1, 4, 8 and 11), six submetacentric pairs (2, 3, 5, 6, 12 and 13), and three subtelocentric pairs (7, 9 and 10). A secondary constriction was detected in the pericentromeric region of the short arm of the homologs of pair 9 in *P. ayeaye* and *P. palliatus*, although in the *P. megacephalus* karyotype, the secondary constriction was observed in the pericentromeric region of the long arm of the homologs of pair 8. Additional secondary constrictions were observed heterozygously in the interstitial region of the long arms of chromosomes 3 and 8 in all the individuals analyzed, as well as in the pericentromeric region of the short arm of chromosome 4 (Fig. 1A).

The heterochromatin revealed by the C-banding was arranged in centromeric blocks in the karyotypes of all three species studied here (Figs 1B, 2B and 3B). In *P. ayeaye*, we detected C-positive bands in the pericentromeric region of the long arm of pairs 6 and 8, and in the short arm of pair 11 (Fig. 1B). In *P. ayeaye* karyotype C-banded chromosomes were sequentially stained with DAPI and MM fluorochromes to reveal the A:T and C:G richness and resulted in brilliant signals in regions coincident with heterochromatic blocks detected by C-banding (Fig. 1C). We also detected MM-positive fluorescence signals that coincided with the secondary constrictions observed by conventional staining (Fig. 1E).

In all the karyotypes, the secondary constrictions revealed by conventional Giemsa staining coincided with the NOR sites detected by the Ag-NOR method. In *P. ayeaye* the NORs were detected in the pericentromeric region of the short arm of the both homologs of pair 9 (Fig. 1D), besides of the additional sites in the interstitial region of the long arm of chromosomes 3 and 8 and in pericentromeric region of the short arm of chromosome 4 (Fig. 1D). The additional sites (pairs 3, 4 and 8) were found in all the individuals analyzed, invariably in the heterozygous condition. The FISH assays realized in *P. ayeaye* confirmed additional NOR sites in the pair 9 (Fig. 1F), which are MM-positive, as is typical of the anuran chromosome. In the *P. palliatus* the NOR sites also were detected in the pericentromeric region of the short arm of the homologs of pair 9 (Fig. 2C). Already in *P. megacephalus* the NORs were located in the pericentromeric region of the long arm of the homologs of pair 8 (Fig. 3C) and additionally in one homologue of pair 13 (Fig. 3C).

## Discussion

### Karyotype conservation in the subfamily Phyllomedusinae

The analysis of the chromosomes of the three *Pithecopus* species, presented here, reinforces the conclusion that the macrostructure of the karyotypes of the members of this genus (diploid number and chromosome morphology) is highly conserved (Barth et al. 2009; Bruschi et al. 2013, Bruschi et al. 2014). The extreme homogeneity of these karyotypes allows for the proposal of a number of different hypotheses on the interspecific chromosomal homologies found in the genus. To begin with, the presence of 26 chromosomes in *Pithecopus* represents the plesiomorphic condition in the subfamily Phyllomedusinae (Schmid et al. 1995, Morand and Hernando 1997, Gruber et al. 2013, Bruschi et al. 2014b, Barth et al. 2014, Schmid et al. 2018). Currently, this subfamily assemble 65 species distributed in eight genus (*Agalychnis* Cope, 1864, *Callimedusa* Duellman, Marion & Hedges, 2016, *Cruziohyla* Faivovich, Haddad, Garcia, Frost, Campbell & Wheeler, 2005, *Hylomantis* Peters, 1873 “1872”, *Phasmahyla* Cruz, 1991, *Phrynomedusa* Miranda-Ribeiro, 1923, *Phyllomedusa* Wagler, 1830, *Pithecopus* Cope, 1866) and only 22 species have been karyotyped (Perkins et al. 2019). The karyotype of the phyllomedusines is highly conserved (Barth et al. 2013; Gruber et al. 2013; Bruschi et al. 2014; Schmid et al. 2018). The unique variation in chromosome morphology found in the species of the genus *Phyllomedusa* karyotype, in particular in the *P. tarsius* group (*P. camba* De la Riva, 1999, *P. tarsius* Cope, 1868, *P. neildi* Barrio-Amorós, 2006, and *P. trinitatis* Mertens, 1926), with three telocentric chromosome pairs (pairs 7, 10, and 12), may represent a possible synapomorphy in this group (Bruschi et al. 2014b).

Like the other species of the genus *Pithecopus* (Bruschi et al. 2012, 2013, 2014), the heterochromatin in *P. palliatus* and *P. ayeaye* is found essentially in the centromeric regions of the all chromosomes, with no distinct band or other marking that permits the differentiation of the karyotypes. The only *Pithecopus* species that can be distinguished based on its C-banding pattern is *P. nordestinus*, which is characterized by a considerable accumulation of heterochromatin, primarily in centromeric regions, extending to the pericentromeric portions of both arms of the chromosome 9 (Bruschi et al. 2012), which is a characteristic of this species.

### Multiple rDNA sites in the karyotype of Pithecopus

The extreme chromosomal conservation observed in the *Pithecopus* species contrasts with its considerable inter- and intrapopulation variation in the chromosomal pairs that carry the 28S rDNA gene clusters. In the present study, two new cases of multiple NOR sites were recorded in the genus *Pithecopus*, in the karyotypes of *P. ayeaye* and *P. megacephalus*. However, a comparative overview of all the *Pithecopus* karyotypes analyzed up to now indicates the recurrence of the NOR-bearing chromosome pairs, and the position of the NORs on these chromosomes, in particular in pairs 3, 4, 8, 9, 11, and 13. Multiple NORs are common in this genus, and have been recorded in practically all the species (Morando and Hernando 1997, Barth et al. 2009, 2013, Paiva et al. 2009, Bruschi et al. 2012, 2013 and present study). In most cases, the karyotypes shown a NOR-bearing pair (homozygosis), detected in all specimens of population whereas the additional NOR-sites occurred in heterozygous and polymorphic condition (Morando and Hernando 1997; Barth et al. 2009, 2013; Paiva et al. 2009; present study). Although intrapopulation variation in the number of NORs is a frequent condition in anuran species, the configuration found in *Pithecopus* reflects the unique evolutionary dynamics of this chromosomal marker.

**Figure 4.**
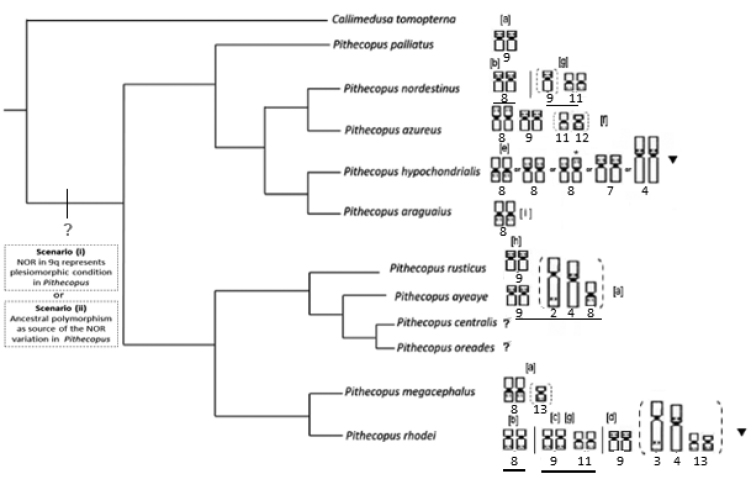
The active NOR-bearing chromosomes found in the karyotypes of the *Pithecopus* species and the broader phylogenetic context of the genus. Two possible scenarios to explain NOR variation are shown in inset (see details in discussion). The phylogenetic arrangement was reconstructed from Duellman et al (2016) and Haga et al. (2017). Chromosomes within brackets present additional NOR sites in the polymorphic condition within the population. The NOR site of the underlined pairs (black lines) was confirmed by FISH using the rDNA probe. Species with unknown karyotypes are indicated by the “?” symbol. Species suspected to contain cryptic diversity are represented by triangles. The letters within brackets indicate the following references: [a] Present study; [b] Bruschi et al. (2012); [c] Barth et al. (2009); [d] Paiva et al. (2009); [e] Bruschi et al. (2013); [f] Morand and Hernando (1997); [g] Barth et al (2013); [h] Bruschi et al. (2014a); [i] karyotype described by Bruschi et al. (2013) and recognized as a new species by Haga et al. (2016). The asterisks (*) represent the heteromorphic condition resulting from the paracentric inversion found in the Alta Floresta population by Bruschi et al. (2013).

The interesting feature of the genus *Pithecopus* is that when the polymorphic condition is recorded in the different species, it to be located in the same chromosomes and NOR positions. Thus, it is difficult to recognize the phylogenetic signal of this marker for the application of a parsimonious evolutionary analysis. Here, we suggest two possible scenarios to explain this variation: (i) the NOR in pair 9q represents the plesiomorphic condition in *Pithecopus*, with subsequent rearrangements resulting in the repositioning of the NOR to pair 8 in *P. azureus* and in the ancestor of *P. hypochondrialis* + *P. araguaius*, with the NOR in pair 8q also representing an autapomorphy in *P. megacephalus*. Subsequent independent events of the loss or gain of rDNA would have resulted in the appearance of the rDNA sites in chromosomes 3, 4, 7, 11, and 13 in the species with the polymorphic condition. In this context, the NOR in pair 9q should be present in the most recent common ancestor (TMRC) of the *Pithecopus* genus (see Figure 4). Alternatively (ii) an ancestral polymorphism would be the source of the extreme variation in the NOR found in this genus.

While the first of these explanations depends on high rates of loss/gain of copies of the rDNA in the genomes of the species, the second hypothesis would depend on the recurrence of the same pairs as the NOR-bearing chromosomes in the different species in the genus *Pithecopus* (see Fig. 4), which would be consistent with the idea of an ancestral polymorphism as the source of the complex scenario observed in the present day. If this hypothesis is accepted, any attempt to trace an evolutionary pathway from this chromosomal marker will inevitably generate a high degree of homoplasy in the phylogenetic inferences, which is typical of the multiple paralogous copies of this marker in the genome (Robinson et al. 2008).

Assuming the ancestral polymorphism hypothesis, the total reproductive isolation of each evolutionary lineage would have resulted in the fixation of the principal active NOR sites in at least one pair of homologous chromosomes (the homozygous condition), which would permit the degeneration of the other sites, or at least the reduction or silencing of their expression. In *P. nordestinus* and *P. ayaye*, respectively, the position of the active NOR detected by Ag-NOR was confirmed by the FISH using 18S/28S rDNA probes (Barth et al. 2013 and present study), which is consistent with the observation of a homozygous principal pair, together with additional, heterozygous sites, that bear the rDNA gene. While a cell requires at least one cluster of active rDNA to satisfy its demand for ribosomal RNAs, there does not appear to be any restriction on the maximum number of copies in a genome (Cazaux et al. 2011). The case of the species of the genus *Mus* is an example of this, in which 1–21 clusters of the rDNA are found in a given karyotype (Cazaux et al. 2011). Given this, not all rDNA sites are being expressed in the cells, and some may be silenced or even lost during the diversification of the different lineages (e.g., Derjusheva et al. 1998; Santos et al. 2002). The number, chromosomal distribution and inheritance of NOR are an important character to genome comparison in Anuran genomes, as observed in water frogs *Pelophylax lessonae* Camerano, 1882, *Pelophylax ridibundus* Pallas, 1771 and in their natural hybrids (*Pelophylax esculentus* Fitzinger, 1843) (Zalesna et al. 2017). In this case, active NOR variability are relationships with ploidy level in hybrids and denote the intragenomic behavior of this chromosomal marker.

One particularly illustrative example of this scenario is the variation in *P. hypochondrialis* found by Bruschi et al (2013), who detected a pronounced population structure based on the analysis of fragments of mitochondrial and nuclear genes. This study found clear differences among populations, and geographical coherence between the clades recuperated by phylogenetic analysis and the NOR-bearing chromosome, which indicates the possible fixation of distinct chromosomes that bear the transcriptionally-active rDNA genes in populations connected by little gene flow. The principal NOR-bearing chromosomes in this species were pairs 4, 7, and 8, in addition to a polymorphic population with extra sites in pairs 3 and 4. This regional chromosomal variation may reflect the role of population dynamics in the fixation of the active NOR from the pool of rDNA sites present in the ancestral genome. Once fixed one chromosome pair with NOR site at a population level, the additional copies of rDNA may either (i) become free of selective pressure and degenerate through stochastic events which would account for the absence of hybridization signals in the FISH experiment or (ii) remain silenced in genome and for consequence undetectable by Ag-NOR method. It is important to note here that Bruschi et al (2013) did not design the experiment to evaluate these specific questions.

The results of the present study also indicate clearly a predominance of rDNA sites located in the pericentromeric and/or subterminal regions of the chromosomes (Fig. 4). Similar results have been obtained for many examples in Anuran karyotypes, as observed in species of the hylid tribe Cophomantini (see Ferro et al. 2018) or in species of the *Agalychnis* Cope, 1864 and *Scinax* Wagner, 1830 genus (Schmid et al. 2018), for example. A number of studies indicate that the NOR-bearing sites in the chromosomes act as hotspots of chromosomal rearrangement (Cazaux et al. 2014). The mechanisms recognized traditionally include the occurrence of unequal crossovers, ectopic recombination, and invasion by mobile genetic elements, all of which have been invoked to account for the observed variation and dispersal of the copies of the NOR in the genome (Poletto et al. 2010; Cazaux et al. 2011; Silva et al. 2013). The evidence points to the possible occurrence of intrachromosomal rearrangements (peri- and paracentric inversions) as the source of the variation in the position of the NOR, such as the distinct positions (8p and 8q) that the rDNA site occupies in the homologs of pair 8 in the different populations of *P. hypochondrialis* (see Fig. 4), for example.

## Conclusions

Our findings help to fill the gaps in the knowledge of the karyotype variability of the genus *Pithecopus* and constitute a good example of the complex role of the rDNA genes in karyotype evolution. Ours results reveals that evolutionary dynamics of the NOR sites in genus and its potential as hotspot of chromosomal rearrangements, which implies that it may be a fundamental feature of chromosomal evolution in the genome of *Pithecopus*.
